# Flat-pressed wood plastic composites from sawdust and recycled polyethylene terephthalate (PET): physical and mechanical properties

**DOI:** 10.1186/2193-1801-2-629

**Published:** 2013-11-23

**Authors:** Khandkar- Siddikur Rahman, Md Nazrul Islam, Md Mushfiqur Rahman, Md Obaidullah Hannan, Rudi Dungani, HPS Abdul Khalil

**Affiliations:** Forestry and Wood Technology Discipline, Khulna University, Khulna, 9208 Bangladesh; School of Life Sciences and Technology, Institut Teknologi Bandung, Gedung Labtex XI, Jalan Ganesha 10, Bandung, 40132 West Java Indonesia; School of Industrial Technology, Universiti Sains Malaysia, 11800 Penang, Malaysia

**Keywords:** Hot pressing, Recycling, Plastic wastes, Modulus of rupture, Modulus of elasticity

## Abstract

This study deals with the fabrication of composite matrix from saw dust (SD) and recycled polyethylene terephthalate (PET) at different ratio (w/w) by flat-pressed method. The wood plastic composites (WPCs) were made with a thickness of 6 mm after mixing the saw dust and PET in a rotary type blender followed by flat press process. Physical i.e., density, moisture content (MC), water absorption (WA) and thickness swelling (TS), and mechanical properties i.e., Modulus of Elasticity (MOE) and Modulus of Rupture (MOR) were assessed as a function of mixing ratios according to the ASTM D-1037 standard. WA and TS were measured after 24 hours of immersion in water at 25, 50 and 75°C temperature. It was found that density decreased 18.3% when SD content increased from 40% to 70% into the matix. WA and TS increased when the PET content decreased in the matrix and the testing water temperature increased. MOE and MOR were reached to maximum for the fabricated composites (2008.34 and 27.08 N/mm^2^, respectively) when the SD content were only 40%. The results indicated that the fabrication of WPCs from sawdust and PET would technically feasible; however, the use of additives like coupling agents could further enhance the properties of WPCs.

## Introduction

Wood plastic composites (WPCs) are relatively new generation of composite materials and also the most promising sector in the field of both composite and plastic industries. In 1970s, the modern concept of WPC was developed in Italy and gradually got popularity in the other part of the world (Pritchard 2004). Wood in the form of flour/particles/fibers are combined with the thermoplastic materials under specific heat and pressure for producing WPCS where additives are added for improving the quality. Many researchers have been worked on WPCs by flat-pressed method at various wood-plastic ratio (Chen et al. 2006; Najafi et al. 2007; Lee et al. 2010; Ayrilmis et al. 2011; Ayrilmis and Jarusombuti 2011; Jarusombuti and Ayrilmis 2011) which typically ranges between 50 to 80% of SD or fibre either as filler or reinforcements (Clemons 2002). The higher strength and aspect ratio of natural fibres offers good reinforcing potential in composite matrix compared to the artificial fibres (Abdul Khalil et al. 2014; Clemons 2008).

Virgin plastics include high and low density polyethylene (HDPE and LDPE respectively), polypropylene (PP), polystyrene (PS) and poly vinyl chloride (PVC) which are commonly used for the production of WPCs (Najafi et al. 2007). Recycled plastics can also consider for manufacturing of WPCs depending on their melting temperature (Stark et al. 2010). Additives can also be added to improve the quality of the composites by eliminating the off-putting properties. However, the utilization of recycled plastic in WPC manufacturing is still limited, and a major portion of global municipal solid waste includes post consumer plastic materials like HDPE, LDPE, PVC, and PET which have the potentiality for being used in the WPCs (Chaharmahali et al. 2008). These post consumer plastics also pose a serious threat to the environment unless they are recycled.

Polyethylene terephthalate commonly known as PET and is formed from terephthalic acid (TPA) and ethylene glycol (EG). It is a long-chain polymer (C_10_H_8_O_4_)_n_ belongs to the polyesters family. It shows both amorphous (transparent) and semi-crystalline nature (Ozalp 2011). PET is intensively used by the packaging industries for bottles and containers of food and other consumer products. Later, PET has started to use in injection molded and extruded articles, primarily for reinforcement with glass fibre (Sinha et al. 2010) which do not degrade in the outdoor environment. Thus, increasing interest has recently been focused on the recycling of plastic wastes, especially PET for these various purposes which could prevent the environmental pollution. Saw dust, a waste from wood processing industries, also creates environmental hazard unless reprocessed for different applications like particleboard, pulp. The recycled PET and saw dust can be used to produce wood plastics by flat-press method which might a good value added products from waste and would help to minimize the waste. Flat press method is newly introduced method in the WPC sector and is similar to the industrial particleboard manufacturing process. Though extrusion and injection molding are the predominant technologies to produce WPCs, but flat press process is technically more advantageous (Jarusombuti and Ayrilmis 2011). This technology possesses some advantages like higher productivity with relatively lower pressure requirement and as a consequence naturally given wood structure lefts undestroyed. Thus, the density of WPCs reduces considerably (Ayrilmis and Jarusombuti 2011; Jarusombuti and Ayrilmis 2011) and increase the moisture resistance properties compared to the conventional wood based composites (Jarusombuti and Ayrilmis 2011). However, there is very limited/no work so far on the fabrication and properties of flat-pressed WPCs from sawdust and recycled PET at various mixing ratios. Thus, the purpose of this study was to investigate the feasibility of wood plastic composites fabrication from sawdust and PET. Determining the physical and mechanical properties of WPCs as a function of mixing ratio was also an objective of this study.

## Materials and methods

### Preparation of raw materials

Sawdust was obtained from the local saw mills in Khulna, Bangladesh. Sawdust was screened to remove the impurities. It was then dried in an oven at 103 ± 2°C for 24 hours for a moisture content of 2%. Clean consumer drinking water bottles were collected locally and grind in a grinder for getting the recycled PET powder. The PET powder was sieved by 60 mesh size sieve to remove the oversized particles. The PET powder was then dried in an oven at 103 ± 2°C for 24 hours for a moisture content of 3% or less. Density, melt flow index and melting point of recycled PET was 1370 (Kg/m^3^), 18.4 g/10 min and 260°C, respectively.

### Flat-pressed sawdust-PET composite manufacturing

The ensuing sawdust and PET powder were mixed for 6 minutes in a rotary drum type blender according to the ratio of Table 1 for producing a homogenous composite. WPC panels were manufactured by flat press process using a dry blending method which was similar to an industrial production process. The mixture was placed in an aluminium caul plate using a forming box to form uniform mat. The press cycle consisted of three phases (Chen et al. 2006), i.e., first phase involved the manual pressing to reduce the mat height, second phase involved in shifting it to the electrically heated improvised hot press for hot pressing, and finally for cold pressing to facilitate the setting of thermoplastic resin. The maximum pressing temperature, pressure, time and cold pressing or pressure holding time were 190°C, 5 N/mm^2^, 25 minutes and 6 minutes, respectively. Lower temperature (190°C) compared to melting temperature (260°C) of the PET was set to avoid the degradation of the wood components. After cold pressing, the WPCs were removed from the press for further cooling. At least six replications of each type of WPC panels having 30 × 25 × 0.6 cm dimension were fabricated. The WPC panels were then trimmed and put into a conditioning room before testing for 48 hours.

**Table 1:** **Formulations of sawdust-PET composites**

Formulation	WPC composition based on % weight	
	Sawdust content (%)	PET content (%)
**SD-40**	40	60
**SD-50**	50	50
**SD-60**	60	40
**SD-70**	70	30

### Evaluation of composite properties

For both physical and mechanical properties, room temperature and relative humidity was 23 ± 2°C and 65 ± 2%, respectively. According to the ASTM standard D-1037 (ASTM 1999), all specimens were carefully prepared and tested to evaluate the physical and mechanical properties of each type of WPCs. At least 24 specimens from 6 replications were used for each type of WPC panel for the evaluation of physical and mechanical properties. The results were compared with the wood based panels as there was no WPC panel standard for comparison as were reported by Ayrilmis et al. (2011).

#### Physical properties

Density was measured according to the standard for composites. Moisture content of WPC panels was measured by oven dry method by using the equation 1:1

Where, mc is the moisture content, m_int_ is mass with moisture (g) and m_od_ is mass after drying.

After soaking the samples in water for 24 hours at 25, 50 and 75°C, water absorptions and thickness swelling were measured according to Najafi et al. (2007). The water absorption (A) and thickness swelling (G) of the specimens were calculated as percentage. The water absorption (A) and thickness swelling (*G*) was calculated according to the Equation 2 and 3, respectively:2

Where m_2_ is the weight (g) of the specimen after soaking and m_1_ is the weight (g) of the specimen before soaking.3

Where, A_1_ is the thickness before soaking, and A_2_ is the thickness after soaking.

#### Mechanical properties

WPC panels were cut into rectangular sections for determining MOE and MOR. The dimension of the specimen was 240 mm × 50 mm × 6 mm. MOE and MOR were measured by following the three point bending test using universal testing machine IMAL-IB600 according to the ASTM D 1037–93 standard (ASTM 1999).

### Statistical analysis of data

Statistical analysis was done by using SAS system software (version 6.12) at 95% confidence level. The significance of different treatments was determined by least significant difference (LSD) test.

## Results and discussion

### Physical properties

#### Density

**Figure 1:**
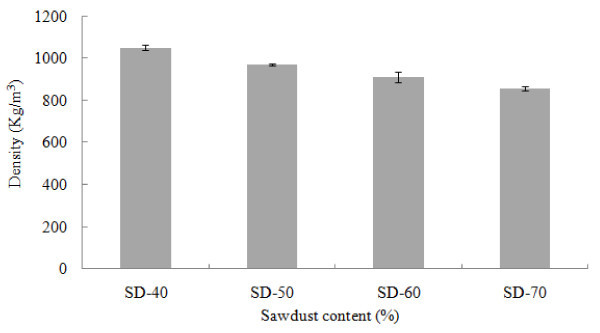
**Density of SD-PET composites at varying ratio.**

The most important indicator of composite’s performance is density, which basically affects all the properties of composites. Density of the SD-PET composites decreased with the increase of SD percentage in the thermoplastic matrices. The lowest density (856.73 Kg/m^3^) was found in composites made with a mixing ratio of 70:30 (SD:PET), whereas the highest density (1048.55 Kg/m^3^) was found when the ratio was 40:60 (SD:PET) (Figure 1). The lower density of sawdust compared to PET might be the cause of this density reduction. Statistical analysis illustrated significant differences (α = 0.05) for density among the SD-PET composites for different mixing ratio (Table 2). According to ANSI (1999) standard, the density of high density particleboard is above 800 Kg/m^3^. Hence, the density of SD-PET composites was above that required standard for high density particleboard. Chen et al. (2006) reported that the smaller wood particles like sawdust would make a thinner mat and the compaction ratio would be higher resulting high density composite materials. That might be another reason for this higher density of WPCs found in this study.

**Table 2:** **Effect of sawdust content on the properties of SD-PET composites**

WPC panel type	Physical properties								Mechanical properties	
	Density (Kg/m^3^)	MC (%)	WA (%)			TS (%)			MOE (N/mm^2^)	MOR (N/mm^2^)
			25°C	50°C	75°C	25°C	50°C	75°C		
**SD-40**	1048.55^A^	0.92^D^	13.8^D^	21.5^C^	23.3^C^	5.7^C^	6.42^A^	7.29^D^	2008.34^A^	27.08^A^
	(30.53)	(0.21)	(1.46)	(3.65)	(2.04)	(0.15)	(0.32)	(0.28)	(107.1)	(5.41)
**SD-50**	968.99^B^	1.37^C^	16.7^C^	24.4^C^	30.2^B^	8^B^	8.68^B^	9.18^C^	1892.91^B^	22.99^B^
	(11.21)	(0.1)	(0.73)	(0.68)	(0.84)	(0.7)	(0.29)	(0.4)	(55.19)	(2.02)
**SD-60**	912.30^C^	1.73^B^	21.3^B^	29.4^B^	33.8^B^	8.1^B^	9.55^C^	10.1^B^	1729.96^C^	14.60^C^
	(59.72)	(0.23)	(1.09)	(2.74)	(8.13)	(0.71)	(1.13)	(0.68)	(35.49)	(4.56)
**SD-70**	856.73^D^	2.15^A^	29.5^A^	36.1^A^	40.3^A^	10^A^	10.9^D^	12.4^A^	1433.93^D^	11.68^D^
	(20.5)	(0.19)	(0.59)	(2.19)	(2.34)	(0.4)	(0.31)	(0.73)	(94.11)	(1.07)

Values in parenthesis are standard deviation.

Values within the same line column by different letters are significantly different at α = 0.05.

#### Moisture content

**Figure 2:**
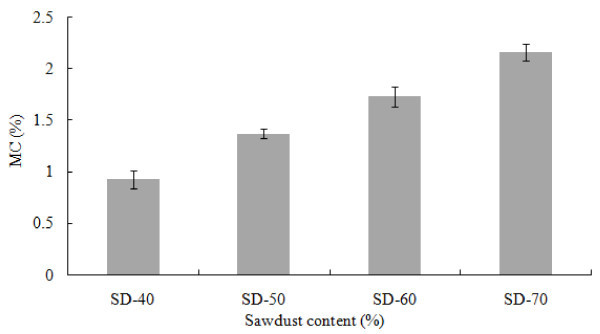
**Moisture content of SD-PET composites at varying ratio.**

The moisture content of the SD-PET composites increased along with the increase of SD percentage from 40 to 70% (Figure 2). Adding SD into the thermoplastic matrix increased the moisture content due to the hydrophilic nature of wood. Additionally, the gaps and flaws at the interfaces, and the micro-cracks in the matrix formed during the manufacturing process boosted up the moisture content as reported by Adhikary et al. (2008). From the variance analysis and LSD (Table 2), it was observed that there was significant difference (α = 0.05) in moisture content among composites. According to the ANSI (1999) standard, the mean moisture content of the board shall not exceed 10% (based on the oven dry weight of the board). The moisture content of SD-PET composites was substantially lower than that of the required standard. The same increasing pattern of MC was also reported by Chen et al. (2006) for the thermoplastic composites made from HDPE and recycled wood particles.

#### Water absorption

**Figure 3:**
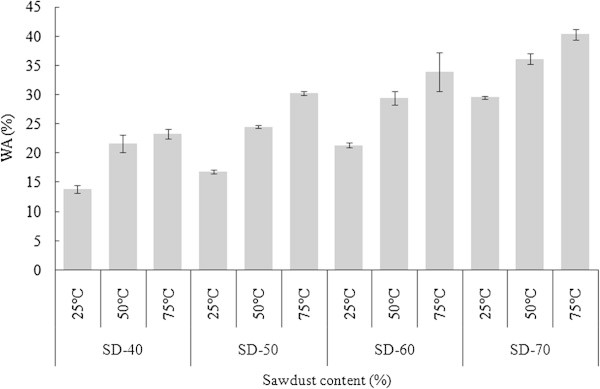
**Water absorption of SD-PET composites at varying ratio.**

Figure 3 illustrates the water absorption of the SD-PET composites based on various SD content and different temperatures (25, 50 and 75°C) after 24 hours of immersion in water. WA of the composites increased with the increase of SD content. The highest WAs were 29.52, 36.10 and 40.33% for 25, 50 and 75°C temperature, respectively with 70% SD content. These results mainly attributed due to the hydrophilic nature of wood. Wood is a hydrophilic porous composite which consists of cellulose, lignin and hemicellulose polymers that are rich in functional groups such as hydroxyls, which readily interact with water molecules by hydrogen bonding (Clemons 2002) and due to this reason, the WPCs have the potentiality to uptake water under humid condition (Adhikary et al. 2008). Similar results for increasing pattern of WA were also reported by Chen et al. (2006) for the WPCs made from HDPE and recycled wood particles. On the other hand, higher water resistance of composites with the increasing PET content can be attributed to the hydrophobic character of PET, though it is semi-crystalline in nature. Figure 3 also illustrated the water uptake as a function of temperature. Immersion temperature had also significant influence on the WA of the composites. In composites with higher SD contents, WA increased more rapidly when the temperature increased from 25°C to 75°C. The trend was reverse for composites having lower SD content. Statistical analysis showed that there was significant difference (α = 0.05) in WA after 24 hours at three different temperatures (25, 50 and 75°C) among the SD-PET composites (Table 2). Najafi et al. (2007) reported that besides the percentage of wood flour/particle, there were several factors including plastic type, virginity of the plastic and surrounding temperature also influenced the water absorption of WPCs.

#### Thickness swelling

**Figure 4:**
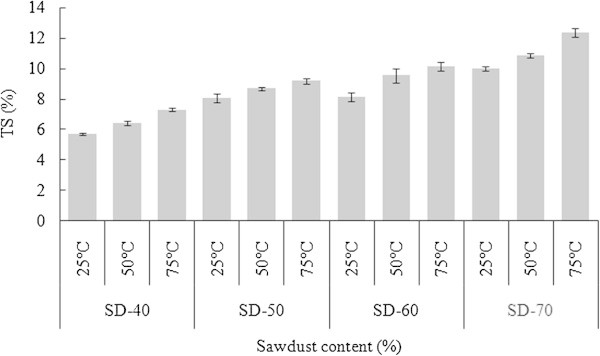
**Thickness swelling of SD-PET composites at varying ratio.**

The tendency of TS was similar to the WA (Figure 4). From the statistical analysis (Table 2), it was observed that the TS were significantly different (α = 0.05) for WPCs at three different temperatures (25, 50 and 75°C). It also illustrated that the SD-PET composites having lower percentage of PET were more susceptible to the thickness swelling than those of panels having higher PET content. This might be due to the increasing SD content in the WPC formulation. Ayrilmis et al. (2011) reported that for thickness swelling and water absorption of WPCs, wood fibers were mainly responsible. The TS at room temperature (25°C) ranged between 5.7 and 10.0% for composites made from 40-70% SD content. The lowest thickness swelling was found for SD-40 composites which might be because of the higher compatibility between SD and PET when compared to the other formulations. This increasing pattern of TS was similar to the results of TS stated by Chen et al. (2006) for the thermoplastic composites made from HDPE and recycled wood particles. Wood has a critical surface energy in the range of 40–60 mJ/m^2^ (Gupta et al. 2007) which is higher than that of PET. The large difference in surface energy between PET and wood might make the PET to be water repellent or hydrophobic.

### Mechanical properties

#### Modulus of elasticity (MOE)

**Figure 5:**
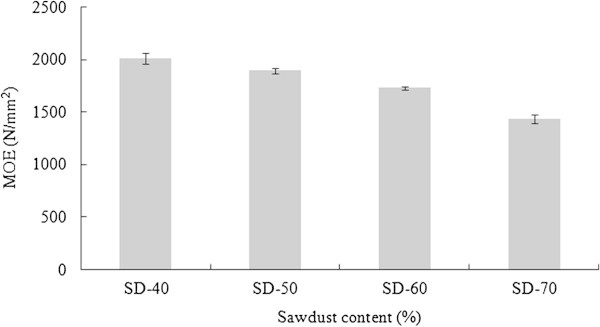
**MOE of SD-PET composites at varying ratio.**

The MOE of composites decreased along with higher SD loading from 40 to 70% in the formulation (Figure 5). This variation might be due to the poor interfacial interaction between the sawdust and PET. The melting temperature of PET was 260°C, however, the pressing temperature was 190°C as a result the thermoplastic (PET) might not flow well within the composites. Shibata et al. (2002) reported that the lower MOE of the composites could be mainly attributed to the poor interfacial interaction between the polymeric matrix and wood particle, not allowing efficient stress transfer between the two phases of the material though the modulus of the natural fibers are higher than the polymeric materials. Some other researches with various thermoplastic materials either virgin or recycled indicated that the MOE of the WPCs would increase with the increase of wood content up to 60% and deceased after 60% of wood content. This was because of more than 60% wood particle used to manufacture WPCs, the plastic material could not totally cover fine wood particles (Chen et al. 2006 and Sanadi et al. 2001). Moreover, Maloney (1977) reported that the relatively large surface area of the fine materials might be another cause of strength loss of the composites. The MOE of WPC panels were statistically different according to the ANOVA and LSD (α = 0.05). MOE of the SD-PET composites could not fulfill the required standard of ANSI (1999) for the high density particle board (2400 N/mm^2^) but all the composites except the 70:30 (SD:PET) fulfilled the required standard (1725 N/mm^2^) for medium density particleboard.

#### Modulus of rupture (MOR)

**Figure 6:**
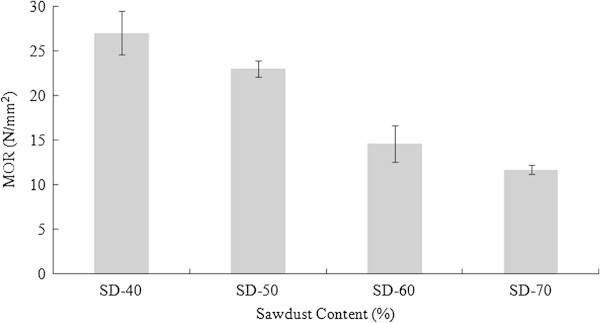
**MOR of SD-PET composites at varying ratio.**

Figure 6 illustrates the effects of SD content on the MOR of the WPCs. The tendency of the MOR was similar to the MOE. It was observed that the MOR of the composites decreased with the increase of SD content and ranged between 11.69 and 27.08 N/mm^2^. It appeared that the binding capacity of the utilized PET gradually decreased. The mechanical behavior of WPCs was greatly influenced by the uniformity of lignocellulosic materials dispersed in the polymeric matrix (Chen et al. 2006 and Raj et al. 1989). The ratio of 40:60 (SD:PET) had the highest MOR value compared to the other formulations. Based on the statistical analysis, significant difference (α = 0.05) was found for the MOR properties of the WPC panels (Table 2). Moreover, only MOR of the SD-40 and SD-50 composites fulfilled the required standard of ANSI (1999) for the high density particleboard (16.5 N/mm^2^). However, all the formulation of composites fulfilled the required standard (11 N/mm^2^) for medium density particleboard. Ayrilmis and Jarusombuti (2011) reported that MOR would increase up to 40–50% for wood fiber content and would start to decrease after 50–60% wood fiber content for flat-pressed PP bonded WPCs.

### Effects of PET content on the properties of composites

Effects of PET content on SD-PET composites are presented in Table 3. Table 3 illustrates that the higher content of PET in the WPC formulation increases the density and bending strength of composites. In the mean time, the increasing PET content in the formulation decreases the moisture content, WA and TS at temperatures of 25, 50 and 75°C. Similar results were also reported by Najafi et al. (2007). It seems that the effect of PET content on moisture content, WA and TS of WPC is positive. Among all the properties of SD-PET composites, PET content showed the most relative effect on WA at 25°C (R^2^ = 0.926), while PET content showed least effect on WA at 75°C for WPCs (R^2^ = 0.693).

**Table 3:** **Effects of PET content on composite properties**

Items	Regression equation	Regression coefficient (R^2^)
**Density (Kg/m** ^**3**^ **)**	y = 6.321x +662.1	0.82
**MC (%)**	y = −0.40x + 3.367	0.865
**WA (%) at 25°C**	y = −0.518x + 43.67	0.926
**WA (%) at 50°C**	y = −0.488x +49.81	0.824
**WA (%) at 75°C**	y = −0.547x + 56.53	0.693
**TS (%) at 25°C**	y = −0.129x + 13.81	0.821
**TS (%) at 50°C**	y = −0.141x + 15.24	0.854
**TS (%) at 75°C**	y = −0.161x + 16.98	0.908
**MOE (N/mm** ^**2**^ **)**	y = 18.86x + 917.4	0.863
**MOR (N/mm** ^**2**^ **)**	y = 0.545x - 5.469	0.751

Significance level α = 0.05; y = Properties of SD-PET composites, x = PET content.

## Conclusion

This study investigated the technical evaluation of flat-pressed wood plastic composites fabricated from different mixing ratios of sawdust and PET. On the basis of physical and mechanical properties, it appears that fabrication of SD-PET composites with dry blending method followed by flat press process is technically feasible for various structural purposes. Therefore, from the above presented results and discussion the following specific conclusions can be drawn:

1. The physical and mechanical property differences among the WPCs are due to the raw material characteristics and the mixing ratios used in the formulations. Therefore, the property of SD-PET composites depends on raw material and mixing ratio.
2. PET contents decreased moisture content, water absorption and thickness swelling of composite. It has also relative effects on density and bending strength.
3. Immersion temperature has significant effect on the water absorption and thickness swelling of WPCs. With the increasing immersion temperature, water absorption and thickness swelling increases.

Though the flat-pressed WPC fabrication from the sawdust and PET is technically feasible, it would be better to mix additives like coupling agents to enhance interaction between sawdust and PET by reducing the melting temperature of PET, and thus, could ensure the adequate physical and mechanical properties of composites.
